# Monitoring Tacrolimus Concentrations in Whole Blood and Peripheral Blood Mononuclear Cells: Inter- and Intra-Patient Variability in a Cohort of Pediatric Patients

**DOI:** 10.3389/fphar.2021.750433

**Published:** 2021-11-05

**Authors:** Amedeo De Nicolò, Michele Pinon, Alice Palermiti, Antonello Nonnato, Alessandra Manca, Jacopo Mula, Silvia Catalano, Francesco Tandoi, Renato Romagnoli, Antonio D’Avolio, Pier Luigi Calvo

**Affiliations:** ^1^ Laboratory of Clinical Pharmacology and Pharmacogenetics, Department of Medical Sciences, University of Turin, Turin, Italy; ^2^ Pediatric Gastroenterology Unit, Regina Margherita Children’s Hospital, Azienda Ospedaliera-Universitaria Città della Salute e della Scienza, Turin, Italy; ^3^ Clinical Biochemistry Unit, Department of Diagnostic Laboratory, A.O.U. Città della Salute e della Scienza Hospital, Turin, Italy; ^4^ General Surgery, Liver Transplant Center, AOU Città della Salute e della Scienza di Torino, University of Turin, Turin, Italy

**Keywords:** pharmacokinetics, immunosuppressant, pharmacology, pharmacokinetics-pharmacodynamics, transplantation, pharmacokinectics, therapeutic drug monitoring

## Abstract

Tacrolimus (TAC) is a first-choice immunosuppressant for solid organ transplantation, characterized by high potential for drug-drug interactions, significant inter- and intra-patient variability, and narrow therapeutic index. Therapeutic drug monitoring (TDM) of TAC concentrations in whole blood (WB) is capable of reducing the incidence of adverse events. Since TAC acts within lymphocytes, its monitoring in peripheral blood mononuclear cells (PBMC) may represent a valid future alternative for TDM. Nevertheless, TAC intracellular concentrations and their variability are poorly described, particularly in the pediatric context. Therefore, our aim was describing TAC concentrations in WB and PBMC and their variability in a cohort of pediatric patients undergoing constant immunosuppressive maintenance therapy, after liver transplantation. TAC intra-PBMCs quantification was performed through a validated UHPLC–MS/MS assay over a period of 2–3 months. There were 27 patients included in this study. No significant TAC changes in intracellular concentrations were observed (*p* = 0.710), with a median percent change of −0.1% (IQR −22.4%–+46.9%) between timings: this intra-individual variability was similar to the one in WB, −2.9% (IQR −29.4–+42.1; *p* = 0.902). Among different patients, TAC weight-adjusted dose and age appeared to be significant predictors of TAC concentrations in WB and PBMC. Intra-individual seasonal variation of TAC concentrations in WB, but not in PBMC, have been observed. These data show that the intra-individual variability in TAC intracellular exposure is comparable to the one observed in WB. This opens the way for further studies aiming at the identification of therapeutic ranges for TAC intra-PBMC concentrations.

## Introduction

Currently, tacrolimus (TAC) is among the most used first-line immunosuppressant drug for the prevention of post-transplantation graft rejection (Staatz et al., 2004; Staatz and Tett, 2004). Despite a rather good effectiveness and tolerability, this drug shows a narrow therapeutic range and quite high intra- and inter-patient variability in its concentrations in whole blood (WB) (Bahmany et al., 2018; Kaneko et al., 2018; Álvarez et al., 2020). For these reasons, therapeutic drug monitoring (TDM) is particularly indicated in order to guide therapeutic adjustments during treatment, particularly in the early phase post-transplantation, and then regularly during the maintenance phase. The current standard matrix for TAC TDM is WB, due to the simple withdrawal, higher concentrations compared with plasma and, consequently, a simpler management in terms of sensitivity. However, TAC is characterized by high and variable protein binding and association with red blood cells (RBC) (Takada et al., 1993; Chow et al., 1997; du Souich et al., 1993). Moreover, as a calcineurin inhibitor, TAC exerts its pharmacological activity within T-lymphocytes (particularly on the Th1 subset (Tron et al., 2019)): therefore, the most useful TDM information would derive from intracellular quantification in lymphocytes (Capron et al., 2011; Lemaitre et al., 2013; Lemaitre et al., 2015; Bahmany et al., 2018). In recent years, this topic deserved some interest and several works described methodological approaches to obtain a reliable quantification of TAC within peripheral blood mononuclear cells (PBMCs) (Capron et al., 2009; Pensi et al., 2015; Pensi et al., 2017; Lemaitre et al., 2020a). On the other hand, information about the clinical usefulness, routine applicability, and intra- and inter-patient variability of TAC intra-PBMC concentration is still poorly explored. Intra-patient variability in drug concentrations represents an important challenge for TDM, since it causes the need for a closer monitoring: particularly, a too high intra-individual variability in the absence of significant clinical or therapeutic changes would limit the usefulness of TDM.

TAC has been shown as characterized by significant intra-individual pharmacokinetic (PK) variability in WB, due to clinical and physiopathological changes in patients’ conditions (such as liver size, function, and regeneration) (Wallemacq et al., 1998; Wallemacq and Verbeeck, 2001; Pinon et al., 2021) and seasonal variability (Lindh et al., 2011). These physiopathological changes can be particularly marked in the pediatric context. In recent years, high intra-individual variability of TAC concentrations in WB was also described as significantly associated with the risk of graft rejection (Del Bello et al., 2018; Schumacher et al., 2020).

Considering all these issues, the primary endpoint of this study was to evaluate and compare the intra- and inter-patient variability in TAC exposure in WB and PBMC in a cohort of pediatric patients undergoing immunosuppressive maintenance therapy, in the absence of significant adverse events and changes in the therapeutic schedule. The secondary endpoint was to investigate eventual factors associated with intra-individual or inter-individual variability in TAC concentrations in WB and PBMC.

## Patients and Methods

### Patients’ Enrolment and Inclusion Criteria

Pediatric patients undergoing maintenance immunosuppressive therapy after liver transplantation with oral TAC in tablet dosage form were prospectively enrolled in this ethically approved study (Città della Salute e della Scienza ethics committe, protocol N° 00107/2019, 25/09/2020). As a clinical policy at the “Regina Margherita Children’s Hospital” TDM was used aiming at target maintenance TAC levels in WB in a range between 2 and 5 ng/ml: particularly, the lower part of this range (around 3 ng/ml) was preferred in patients without any history of rejection or with a history of adverse events (renal impairment or lymphoproliferative disorders).

Patients’ families, during outpatient visit, were required to fill out a questionnaire in order to assess the therapeutic adherence, concomitant drugs, posology, and quantity of the last dose of TAC. Inclusion criteria comprehended: being in maintenance therapy for at least 1 year; no variations in TAC posology or in the therapeutic schedule (including concomitant drugs) during the study period; no concomitant treatment with everolimus or corticosteroids. The concomitant use of mycophenolate mofetil (MMF) was allowed, considering the absence of significant interaction with TAC. The enrollment was performed using the following exclusion criteria: poor therapeutic adherence; wrong timings for drug intake or assumption with food; vomiting after the last dose intake of TAC; therapeutic changes in TAC posology in the last 2–3 months; rejection episodes or use of steroids in the study period; intake of potentially interfering drugs during the study period. After completing the study questionnaire, healthcare personnel determined the patient’s eligibility for blood withdrawal during outpatient visit. The study consisted of at least 2 consecutive visits, with a time lapse comprised between 2 and 3 months, in order to assess intra-patient variability in hematic and intracellular TAC concentrations in the absence of major changes in therapeutic schedules, dose, and clinical episodes. Some patients had more than two PK evaluations. The evaluation of clinical conditions, hematochemical tests including ALT and GGT, total bilirubin, serum creatinine, and hemochrome was performed during each visit.

### Peripheral Blood Mononuclear Cells Isolation

Blood samples were withdrawn at the end of dosing interval, before the morning dose intake, in order to obtain trough concentration (C_trough_) data, at the Pediatric Gastroenterology Unit, Regina Margherita Children’s Hospital, Turin, and delivered immediately to the Laboratory of Clinical Pharmacology and Pharmacogenetics of the University of Turin, where PBMCs isolation and intracellular TAC quantification were performed. The whole procedure of PBMCs isolation took less than 1 h. PBMCs isolation was performed with CPT® (Cell Preparation Tube), centrifuged at room temperature for 15 min at 1600 × g for 15 min at 25°C. Cell layers were collected with a Pasteur pipette and transferred into a falcon tube, brought to a final volume of 50 ml, and then washed twice in sodium chloride 0.9% solution and centrifuged at 2200 × g for 6 min at 4°C, in order to prevent drug loss. Before the second wash, pellet was treated with 2 ml of ammonium salt solution (130 mM ammonium chloride + 7.5 mM ammonium carbonate) for 1 min to obtain red blood cell lysis. After adjusting the volume again to 40 ml with sodium chloride 0.9% solution, 500 μl of cell suspension was diluted, leading to a final volume of 19.5 ml of Isoton and rate in two beakers. The two aliquots were used for cell count and determination of mean cell volume (MCV) through an automated Beckman Coulter Z2 (Instrumentation Laboratory, Milan, Italy). Four counts for each sample (two for each beaker) were performed. Data were processed by Z2 AccuComp software (version 3.01). To obtain blank PBMCs aliquots, the resulting PBMCs pellet was dissolved with an extraction solution (methanol:water, 70:30 [vol:vol]) to a maximum cell concentration for each aliquot of 12 12×10^6^ cell/mL. The resulting cell lysates were divided in aliquots, and then stored at −80°C.

### Quantification of Tacrolimus Concentrations

TDM of TAC exposure in blood samples was performed through UPLC–MS/MS using Masstrak Immunosuppressants XE kit (CE-IVD marked; “Città della Salute e della Scienza” Hospital, Turin, Italy). Blood sampling in sodium/EDTA vacutainers was performed contextually with sampling for PBMC isolation. Similarly, intraPBMC TAC concentrations were quantified by a UPLC-MS/MS assay coupled with an automated solid phase extraction (SPE), as previously described (Pensi et al., 2015; Pensi et al., 2017). Briefly, the method consisted of cell lysis, sample purification, and TAC concentration by SPE and analysis by positive electrospray ionization. The method was linear in a range between 0.039 and 5 ng, with a lower limit of quantification of 0.019 ng for each PBMC aliquot, with mean accuracy of 100.4% and inter-day coefficient of variation of 6.1%.

The normalization of analytical data obtained from the SPE-UPLC-MS/MS analysis was then performed dividing the obtained absolute TAC amount in each PBMC sample by the number of cells and their MCV, previously determined through the automated cell counter. This normalization allowed to directly compare the intracellular TAC concentrations with the ones in WB.

The observed percent change in TAC concentration in WB and PBMC between the first and second timing was considered as a measure of intra-individual variability, calculated as follows: (TAC concentration at time 2 - TAC concentration at time 1)/TAC concentration at time 1.

### Statistical Analysis

All statistical analyses were performed through Excel and SPSS 27.0 (IBM, Armonk, NY).

Descriptive data have been reported as median and interquartile ranges (IQR). Correlations between continuous data have been evaluated through Pearson or Spearman correlation tests, for normally or not-normally distributed variables, respectively. Differences between categorical groups at the same timing have been tested through the Mann-Whitney or Kruskal-Wallis non-parametric rank tests, while differences between timings in the same patients were tested by Wilcoxon test for coupled samples. Variables which resulted in significantly correlated with TAC concentrations and concentration corrected by dose/weight ratio (C/D/Kg) were tested for their predictive value by univariate and multivariate linear regression model, in the absence of significant co-linearity between the tested independent predictors.

## Results

### Patients’ Characteristics

Among 56 pediatric patients treated with TAC after liver transplantation who were screened, 27 fit the inclusion criteria and have been enrolled in this study. The overall demographic, anthropometric, and clinical characteristics of these patients are resumed in Table 1. Eight patients out of 27 (29.6%) had concomitant MMF treatment.

**TABLE 1:** Summary of patients’ characteristics at the two considered timings. Body mass index (BMI), white blood cells (WBC), aspartate amino-transferase (AST), alanin amino transferase (ALT), gamma glutamil transferase (GGT), C-reactive protein (CRP), calculated glomerular filtration rate (eGFR)

Number of patients: 27 Coupled samples: 43 Sex (Male): 16		
	MEDIAN(IQR) T-1	MEDIAN(IQR) T-2
BMI (kg/m^2^)	26 (13–39)	-
Age (y.o.)	7 (5–12)	-
WBC (10^9^ cells/L)	6.1 (4.2–7.5)	6.0 (4.1–7.8)
Gamma globulins (%)	16.15 (13.95–18.33)	15.6 (14.3–18.1)
Total bilirubin (mg/dl)	0.4 (0.3–0.7)	0.5 (0.3–0.9)
Glucose (mg/dl)	78.0 (73.5–83.3)	78.0 (74.0–81.0)
AST (UI/L)	32.5 (23.0–43.5)	35.0 (29.0–45.8)
ALT (UI/L)	25.0 (17.3–38.3)	26.0 (19.3–64.3)
GGT (UI/L)	12.0 (9.0–23.5)	13.0 (10.0–39.3)
CRP (mg/L)	0.5 (0.2–2.9)	0.4 (0.2–0.8)
Creatinin (mg/dl)	0.4 (0.3–0.5)	0.4 (0.3–0.5)
eGFR (ml/min/1.73 mq)	123.5 (113.0–140.8)	126.5 (111.8–139.5)
Albumin (g/dl)	4.4 (4.2–4.6)	4.4 (4.1–4.5)
Vitamin D (ng/ml)	27.4 (17.5–59.3)	27.6 (21.4–40.1)
Haematocrit (%)	39.8 (36.3–41.4)	40.4 (38.0–41.8)

All the included patients completed at least two consecutive visits without showing adverse events or modifications in their immunosuppressive treatment.

### Intra-individual Pharmacokinetic Variability in Whole Blood and Peripheral Blood Mononuclear Cells

The median TAC intracellular concentrations at the first timing and the second timing were 17.5 ng/ml (10.6–27.7) and 16.0 ng/ml (9.8–32.7), respectively. Similarly, median TAC concentrations in WB were 2.8 ng/ml (1.8–3.3) at the first timing and 2.9 ng/ml (1.7–3.8) at the second timing. These differences between timings resulted in not statistically significant (*p* = 0.710 and *p* = 0.870, respectively). Moreover, the intra-individual percent change in TAC concentrations between timings resulted in comparable in WB and PBMC (*p* = 0.902, Table 2). Similarly, also the variance in the percent changes between timings in intra-PBMC and whole-blood concentrations was not significantly different (*p* = 0.743 by Levene test, indicating a homogeneous variance) and resulted in mutually correlated (R = 0.512, *p* = 0.001). Therefore, intra-individual variations in intra-PBMC concentrations resulted in associated with the variations in WB concentrations. Accordingly, the median percent change in the intra-PBMC/whole-blood TAC concentration ratio between timings was −0.4% (−28.2%–+44.5%; *p* = 0.987).

**TABLE 2:** Summary of median (IQR) concentration parameters observed at the first (T1) and second (T2) PK visit.

	MEDIAN (IQR) T-1	MEDIAN (IQR) T-2	% Change	*p* value
WB TAC conc. (ng/ml)	2.8 (1.8–3.3)	2.9 (1.7–3.8)	−2.9 (−29.4–+42.1)	0.870
Intra-PBMC TAC conc. (ng/ml)	17.5 (10.6–27.7)	16.0 (9.8–32.7)	−0.1 (−22.4–+46.9)	0.710
Intra-PBMC/WB ratio	7.0 (5.0–10.0)	6.3 (4.9–8.5)	−0.4(−28.1–+44.5)	0.987
Norm. WB TAC C/D/kg (ng/mg/kg)	48.1 (35.7–67.9)	49.4 (35.4–87.4)	−0.5 (−31.0–+47.0)	0.912
Norm. Intra-PBMC TAC C/D/kg (ng/mg/kg)	372.6 (182.0–480.4)	354.2 (236.5–523.8)	1.9 (−22.3–+42.1)	0.876
Weight-adjusted TAC Dose (mg/kg/day)	0.05 (0.03–0.09)	0.05 (0.03–0.08)	−2.0 (−8.0–+1.0)	0.998

### Factors Associated With Intra-Individual Variability

Once the variability in TAC concentrations in blood and PBMC was described, the correlations between this variability and percent changes in other clinical, hematochemical, and anthropometric characteristics was evaluated. No significant correlations between percent changes in TAC concentrations, neither in whole-blood and PBMC, were observed with changes in BMI, bilirubin, ALT, albumins, hematocrit, and white blood cells count.

**FIGURE 1:**
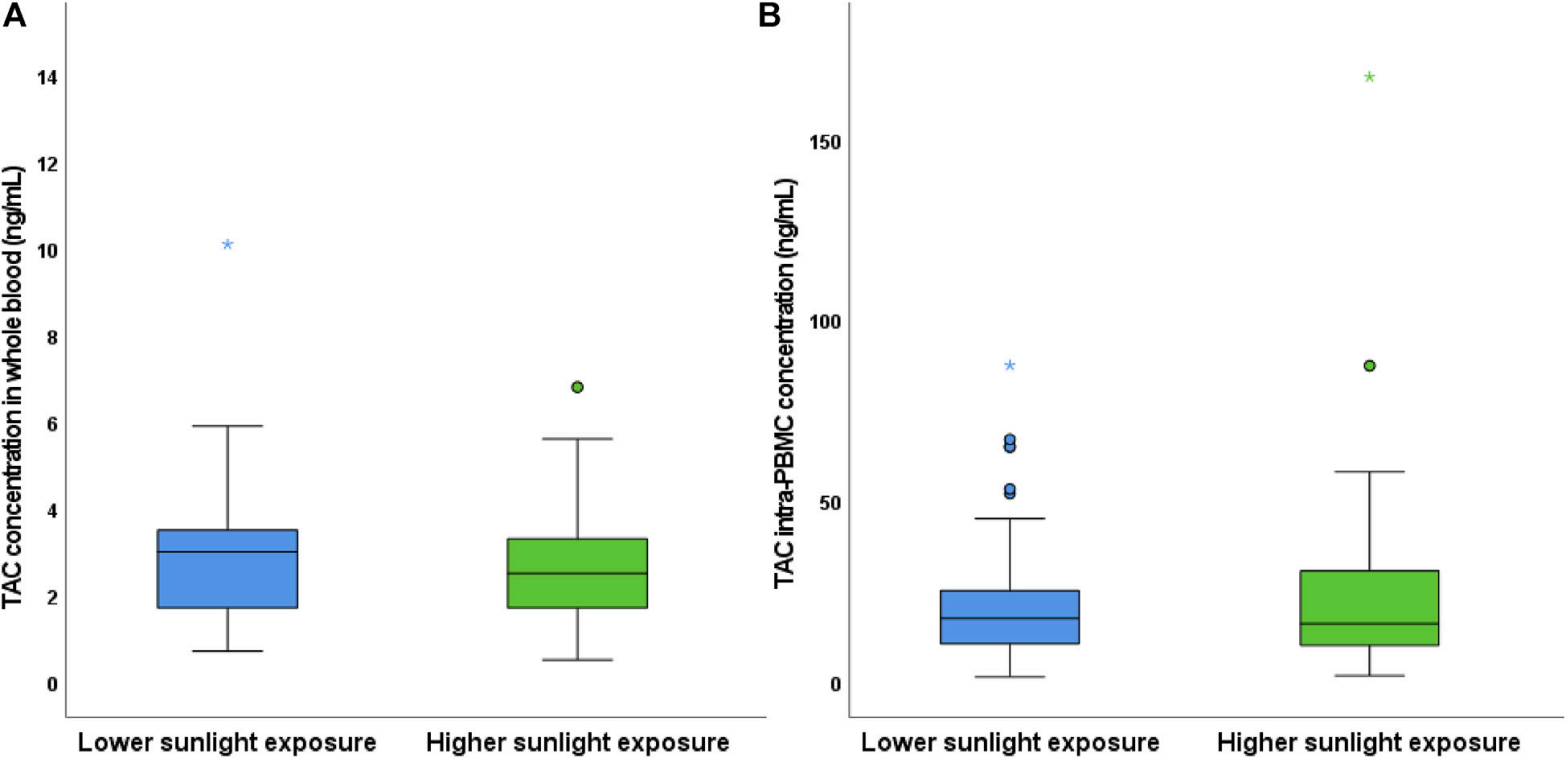
Comparison of TAC concentrations in WB **(A)** and PBMC **(B)** according to seasonal variations. Coupled samples have been assigned to the lower or higher sunlight exposure groups based on the period of the first and second blood sampling (e.g., winter vs autumn/spring or autumn/spring vs summer, respectively).

The percent change in intra-PBMC concentrations between timings appeared associated with borderline significance (R = 0.314; *p* = 0.062) with the minor changes in the weight-adjusted TAC dose (in fact, despite stable TAC dose was administered, patients’ growth accounted for this minimal variability). Deepening this issue, we hypothesized a possible role of seasonal variations in the expression of metabolic enzymes, according to previous data (Lindh et al., 2011). For this reason, for each patient we compared TAC concentrations in WB and PBMC monitored during the periods of minimum and maximum sunlight exposure (winter vs autumn/spring or summer vs spring/autumn): this comparison showed significantly lower TAC concentrations in WB during the periods of higher sunlight exposure (*p* = 0.040, Figure 1), while this difference was not confirmed as statistically significant for intracellular concentrations (*p* = 0.112).

### Inter-individual Variability in Tacrolimus Concentrations

As a secondary endpoint, factors associated with inter-individual variability in TAC concentrations in blood and PBMC among the enrolled patients within the same timing were studied.

A slightly wider inter-individual variability was observed in PBMC, compared with blood, as reported in Table 2. Nevertheless, after normalization by mean values, the variance in the intra-PBMC and WB concentrations was not significantly different at both timings (*p* = 0.263 at time 1; *p* = 0.522 at time 2).

First, TAC concentrations in WB and PBMC were tested for correlation with all patients’ characteristics, showing strongly significant correlations with serum albumins R values and P values are summarized in Supplementary Table S1 (R = −0.384 ; *p* = 0.012 and R = −0.358 ; *p* = 0.019, respectively), hematocrit (R = −0.518; *p* < 0.001 and R = −0.467 ; *p* = 0.002, respectively), gamma-globulinemia (R = −0.348 ; *p* = 0.024 and R = −0.404 ; *p* = 0.007) and, obviously, the weight-adjusted TAC dose (R = 0.636 ; *p* < 0.001 and R = 0.583 ; *p* < 0.001).

Moreover, the weight-adjusted dose showed a significant negative correlation with albuminemia (R = −0.398: *p* = 0.044) and hematocrit (R = −0.750; *p* < 0.001). As expected, considering that the patients were in maintenance treatment and the previous use of TDM for dose adjustments in relationship with TAC toxicity and efficacy, no significant correlations were observed neither with eGFR (R = −0.062; *p* = 0.715 and R = 0.017 *p* = 0.922, respectively) nor with ALT levels (R = 0.042 ; *p* = 0.792 and R = −0.084 ; *p* = 0.593, respectively). No differences were observed in TAC concentrations neither in WB nor in PBMC between patients who had concomitant MMF treatment.

Once the factors correlated with TAC absolute concentration were evidenced, the analysis was refined by testing all the variables for correlation with TAC concentrations adjusted by dose/weight ratio (C/D/Kg), showing a significant correlation with patients’ age (R = 0.301, *p* = 0.056 and R = 0.339, *p* = 0.024 respectively).

### Gender Differences in Tacrolimus Concentrations

**FIGURE 2:**
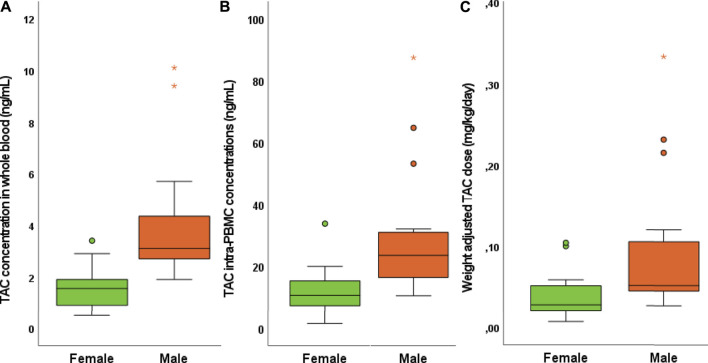
Gender differences in TAC concentrations in WB **(A)**, PBMC **(B)**, and weight adjusted TAC dose **(C)**.

Interestingly, significant gender differences in TAC concentrations were observed both for regarding WB (*p* = 0.003) and PBMC (*p* = 0.002), with male patients showing higher TAC exposure (Figure 2). Conversely, no significant differences between genders were observed in the relative intracellular penetration (*p* = 0.435) and TAC C/D/Kg in blood and PBMC (*p* = 0.124 and *p* = 0.152, respectively). Interestingly, the weight adjusted dose of TAC resulted in significantly higher in male patients (*p* = 0.001), suggesting that the higher TAC exposure in these patients would be due to a previous selection of higher TAC dosage in the male gender.

### Predictive Factors for Tacrolimus Concentrations in Whole Blood and Peripheral Blood Mononuclear Cells

The factors that showed significant associations/correlations with TAC concentrations in WB and/or PBMC and that did not show any significant co-linearity were tested for their predictive power for TAC concentrations in WB and PBMC. By univariate analysis, the weight-adjusted TAC dose, hematocrit, albumin levels, and gender resulted in significant predictors of TAC concentrations in WB (all regression parameters and *p* values are reported in Table 3). Conversely, patients’ age resulted in the only predictor of TAC C/D/kg in WB.

**TABLE 3:** Report of the univariate and multivariate regression analysis for the prediction of TAC concentrations in WB. All the statistics have been reported in APA format.

Whole-blood TAC concentration	Univariate analysis					
		Age	Weight-adjusted dose	Hematocrit	Albumins	Gender
F (d.f.; res.)	-	7.30 (1; 26)	15.80 (1; 26)	5.40 (1; 26)	8.70 (1; 26)
Unstand. B coef.	-	26.30	−0.38	−3.92	2.5
*p* value	-	<0.001	<0.001	0.029	0.007
Intercept	-	1.20	18.20	20.40	1.64
*R* ^2^	-	0.760	0.407	0.190	0.274
**Whole-blood TAC concentration adjusted by dose/weight ratio**	**Univariate analysis**					
		**Age**	**Weight-adjusted dose**	**Hematocrit**	**Albumins**	**Gender**
	F (d.f.; res.)	6.5 (1; 26)	-	-	-	-
	Unstand. B coef.	9.04	-	-	-	-
	*p* value	0.018	-	-	-	-
		Intercept	−9.70	-	-	-	-
	*R* ^2^	0.229	-	-	-	-
**Whole-blood TAC concentration**	**Multivariate analysis (final model)**					
		**Age**	**Weight-adjusted dose**			
	F (d.f.; res.)		63.4 (2; 25)			
	Unstand. B coef.	0.16	28.20			
	*p* value	0.001	<0.001			
	Intercept		−0.342			
		*R* ^2^		0.852			

By multivariate analysis, also considering the presence of significant co-linearity between the weight-adjusted dose, serum albumin, hematocrit, and gender, the only significant mutually independent predictors of TAC concentrations in WB resulted in patients’ age and the weight-adjusted dose (*p* = 0.001 and <0.001, respectively; R^2^ = 0.852).

**FIGURE 3:**
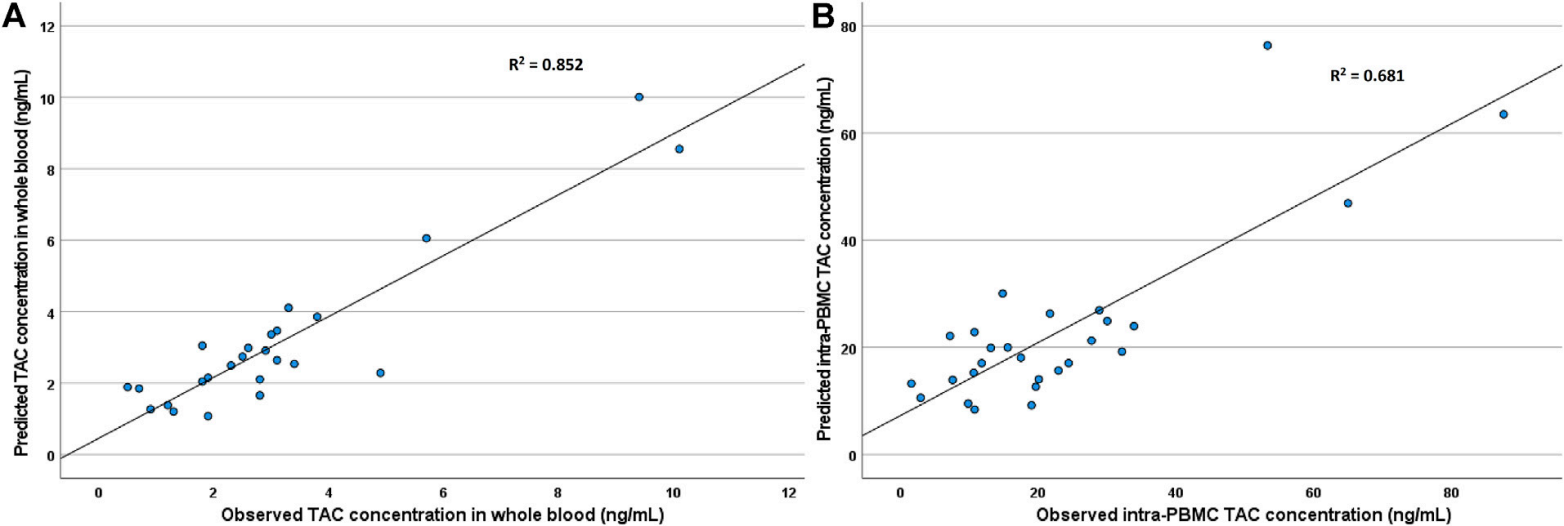
Correlation between the observed TAC concentrations in WB **(A)** and PBMC **(B)** and the predicted concentration obtained by the regression model.

Similar results were observed considering both univariate and multivariate analysis regarding intra-PBMC concentrations (regression parameters are reported in Table 4). Again, the only significant predictors of TAC concentrations in PBMC were patients’ age and the weight-adjusted TAC dose (*p* = 0.049 and <0.001, respectively; R^2^ = 0.681). A graph describing the correlation between the observed TAC concentrations in PBMC and WB and the predicted concentration obtained by the regression model are reported in Figure 3.

**TABLE 4:** Report of the univariate and multivariate regression analysis for the prediction of TAC concentrations in PBMC. All the statistics have been reported in APA format.

Intra-PBMC TAC concentration	Univariate analysis					
		Age	Weight-adjusted dose	Hematocrit	Albumins	Gender
F (d.f.; res.)	-	41.5 (1; 26)	18.3 (1; 26)	5.9 (1; 26)	7.1 (1; 26)
Unstand. B coef.	-	201.2	−3.2	−33.3	17.9
*p* value	-	<0.001	<0.001	0.010	0.013
Intercept	-	8.4	149.3	169.9	12.3
R^2^	-	0.624	0.432	0.190	0.274
**Intra-PBMC TAC concentration adjusted by dose/weight ratio (C/D/Kg)**	**Univariate analysis**					
		**Age**	**Weight-adjusted dose**	**Hematocrit**	**Albumins**	**Gender**
	F (d.f.; res.)	6.8 (1; 26)	-	-	-	-
	Unstand. B coef.	38.0	-	-	-	-
	*p* value	0.015	-	-	-	-
	Intercept	−109.1	-	-	-	-
		*R* ^2^	0.215	-	-	-	-
**Intra-PBMC TAC concentration**	**Multivariate analysis (final model)**					
		**Age**	**Weight-adjusted dose**			
	F (d.f.; res.)	25.7 (2; 25)				
	Unstand. B coef.	−1.0	216.0			
	*p* value	0.049	<0.001			
	Intercept	−1.65				
		*R* ^2^	0.681				

### Association Between Tacrolimus Concentrations and Clinical Parameters

Considering the selection of patients who were in maintenance therapy and no changes in TAC posology during the observation period, no episode of graft rejection in either cases of renal toxicity were observed. A negative significant correlation was observed between TAC concentrations in PBMC and the percentage of gamma-globulins both at the first and second visit (R = 0.393; *p* = 0.012 and R = −0.482; *p* = 0.003, respectively): conversely, only a slightly significant correlation was identified between WB TAC concentration at the second visit (R = 0.337; *p* = 0.049).

No other significant correlations were observed between clinical conditions and TAC concentrations.

## Discussion

In the past years, the TDM of TAC concentrations in WB entered in the common clinical practice for the management of the immunosuppressive treatment after solid organ transplantation (Brunet et al., 2019). This is due to the knowledge of therapeutic ranges associated with low probability of graft rejection and toxicity (Brunet et al., 2019; Lemaitre et al., 2020b). Nevertheless, while this practice has been proved effective for therapeutic optimization, some cases of rejection of toxic effects can be observed at TAC concentrations in WB which fall in the therapeutic range. Recently, some reports showed how TAC concentrations within graft or PBMC could be better predictors of its immunosuppressive effect (Capron et al., 2007; Lemaitre et al., 2013; Lemaitre et al., 2020a).

On the other hand, the adoption of TAC concentrations in PBMC for TDM purposes can be considered only if their intra-individual variability in the absence of changes in posology, concomitant drugs, or other physiological changes is rather contained or at least comparable to the one in WB.

This is particularly important in the pediatric context, since these patients have significantly higher probability to experience adverse events and because of continuous physiological changes due to their growth (Wallemacq et al., 1998; Wallemacq and Verbeeck, 2001; Kearns et al., 2003). For these reasons, this work provided useful information regarding the medium-term changes in TAC concentrations in WB and PBMC, showing that their intra-individual variability can be considered comparable. Moreover, this work confirmed a previous report from Lindh et al. (Lindh et al., 2011) showing that TAC concentrations in WB are affected by seasonal variability, with an inverse trend with sunlight exposure. Nevertheless, no increased incidence of graft rejection was previously reported in literature seasons with higher sunlight exposure or related to vitamin D levels (Mosca et al., 2020): conversely, a single work studying seasonal variation in chronic graft rejection reported a slightly higher incidence in winter (Astor et al., 2017). In our work, we observed significant seasonal changes in TAC concentrations in WB, but not in PBMC, possibly explaining this mismatch between TDM data in WB and clinical outcome.

On the other hand, focusing on the inter-individual variability, we observed that several variables were significantly associated with TAC concentrations in WB and PBMC. Among these, the inter-individual differences in the weight-adjusted dose and age were the only emergent significant predictors of TAC concentrations both in WB and PBMC. Nevertheless, considering the values of the coefficients of determination (R^2^ 0.852 and 0.681, respectively), further 15 and 32% of this variability remains to be explained. Particularly, the lower fit for the intracellular concentrations the involvement of other factors, such as genetic differences in drug transporters (Capron et al., 2010; Lemaitre et al., 2015; Tron et al., 2019; Tron et al., 2020), in the intracellular disposition of TAC.

The observed inverse correlations between serum albumin levels and TAC concentrations, both in WB and PBMC, could be explained by a higher metabolic activity of the liver, which can be associated with increased drug metabolism. On the other hand, the inverse correlation with hematocrit could be explained by the fact that lower TAC dosages could have been selected in patients with higher hematocrit, since TAC adhesion to erythrocytes can reduce its elimination in the initial phases of therapy (Limsrichamrern et al., 2016; Uchida et al., 2020). In this case, TDM-guided posological adjustments during the first year of therapy would be responsible of these differences, in accordance with the observed negative correlation between the weight-adjusted TAC dose and both albuminemia and hematocrit. This hypothesis seems to be confirmed considering the observed gender differences in TAC dose and concentrations, with higher exposure in male patients. In fact, in previous works from our group focusing on the first days of treatment with TAC, a significantly higher TAC concentration was observed in female patients, guiding a dose reduction in these patients in the following days (Calvo et al., 2017; Pinon et al., 2021). Interesting evidence obtained in this cohort was a significant negative correlation between intra-PBMC concentrations and the percentage of gamma-globulins, which was less marked when considering TAC concentrations in WB. This could be interpreted as a stronger immunosuppressive effect in patients with higher TAC concentrations in PBMC.

This study has several limitations: first, no genetic information was available for these patients; moreover, the evaluation of seasonal variability could be better performed by scheduling PK sampling every 3 months in a year, contextually with vitamin D quantification.

Nevertheless, since the primary endpoint was the intra-individual evaluation, no effect of patients’ genetics is expected, while its impact on the inter-individual variability in the absolute TAC concentrations should be contained considering that major dose adjustments guided by TDM, commonly occurring in the first days after liver transplantation, tend to compensate for genetic variability (Pinon et al., 2021). Conversely, regarding the study of seasonal variability in TAC concentrations, this will surely be the subject of future studies.

Concluding, this study shows that the intra-individual variability in TAC concentrations in PBMC and WB are similar, in the absence of significant changes in the therapeutic schedule or in patients’ conditions, making the adoption of intracellular TAC quantification for TDM purpose theoretically possible. Further studies focusing on eventual differences in the predictive power between intra-PBMC and WB concentration for adverse events and graft rejection are needed, in order to assess if a real clinical advantage exists.

## Data Availability

The raw data supporting the conclusions of this article will be made available by the authors, without undue reservation.
