# Supplementary material for: Monitoring Tacrolimus Concentrations in Whole Blood and Peripheral Blood Mononuclear Cells: Inter- and Intra-Patient Variability in a Cohort of Pediatric Patients
Source: Front Pharmacol. 2021 Nov 5;12:750433. doi: 10.3389/fphar.2021.750433 (PMC8602893; doi:10.3389/fphar.2021.750433)
Supplement: Supplementary file 1 [file Table1.docx]

|  | **Whole Blood concentration (ng/mL)**  **[R;P-value]** | | **Intra-PBMC concentration (ng/mL)**  **[R;P-value]** |
| --- | --- | --- | --- |
| **Albumin (g/dL)** | | - .384 ; .012 | - .358 ; .019 |
| **Hematocrit (%)** | | - .518 ; < .001 | - .467 ; .002 |
| **γ-globulinemia (%)** | | - .348 ; .019 | - .404 ; .007 |
| **Wheight-adjusted Tacrolimus dose (mg/Kg/day)** | | .636 ; < .001 | .583 ; < .001 |

**Supplementary table1 (S1):** Correlation between TAC concentrations in WB and PBMC with patient’s characteristics. Statistical parameters have been reported in APA style.
